# Effects of dietary *Chinese yam* polysaccharide copper complex on growth performance, immunity, and antioxidant capacity of broilers

**DOI:** 10.3389/fvets.2023.1123002

**Published:** 2023-02-16

**Authors:** Jinzhou Zhang, Yan Jin, Mengmeng Cao, Jiahua Deng, Yadi Chang, Mingyan Shi, Zhiguo Miao

**Affiliations:** ^1^College of Animal Science and Veterinary Medicine, Henan Institute of Science and Technology, Xinxiang, Henan, China; ^2^Life Science College, Luoyang Normal University, Luoyang, Henan, China

**Keywords:** *Chinese yam* polysaccharide copper complex, growth performance, immunity, oxidation resistance, broilers

## Abstract

*Chinese yam* polysaccharide (CYP) has received attention in recent years owing to its positive nutritional and medicinal characteristics. Copper is an essential trace metal in animals, which plays an important role in iron absorption and hemoglobin synthesis. However, no published study has evaluated *Chinese yam* polysaccharide copper complex (CYP-Cu) as a dietary additive in broilers. This study was conducted to investigate the effects of dietary CYP-Cu on growth performance, immunity, and oxidative resistance in broilers. A total of 360 1-day-old 817 broiler chickens were randomly divided into 4 groups, with 3 replicates of 30 birds each and were fed a basal diet with the addition of 0 (control group), 0.02, 0.10, and 0.50 g/kg CYP-Cu. The feeding trial lasted 48 days. On day 28 and day 48, 6 broilers in each group were slaughtered, respectively. Then the parameters of growth and carcass, serum biochemistry, immunity, and antioxidation, and the expression level of hepatic antioxidative genes were investigated. The results showed that compared with the control group, the supplementation of dietary CYP-Cu could improve the indexes of the growth, carcass, serum biochemistry, immunity and oxidation resistance in broilers, such as average daily gain (ADG), the slaughter percentage (SP), semi-evisceration weight percentage (SEWP), eviscerated carcass weight percentage (EWP), breast muscle percentage (BMP), leg muscle percentage (LMP), serum albumin (ALB), high density lipoprotein (HDL), insulin-like growth factor I (IGF-I), triiodothyronine (T3), thyroxine (T4), growth hormone (GH), insulin (INS), immunoglobulin M (IgM), immunoglobulin G (IgG), immunoglobulin A (IgA), interleukin 2 (IL-2), interleukin 4 (IL-4), interleukin 6 (IL-6), complement 3 (C3), complement 4 (C4), total superoxide dismutase (T-SOD), total antioxidative capacity (T-AOC), glutathione peroxidase (GSH-Px), and glutathione s-transferase (GSH-ST); these parameters in the 0.10 g/kg CYP-Cu treated group were significantly increased (*P* < 0.05) in the total trial period, with the exceptions that feed conversion ratio (FCR) and serum low density lipoprotein (LDL), malondialdehyde (MDA) were decreased in the total trial period. In addition, the antioxidative gene mRNA expression of Nuclear factor E2-related factor 2 (*Nrf*_2_), Superoxide dismutase 1 (*SOD*_1_), Superoxide dismutase 2 (*SOD*_2_), and Catalase (*CAT*) were upregulated in the liver (*P* < 0.05). These results indicated that the supplementation of dietary CYP-Cu improved the growth, immunity, and oxidation resistance of broilers, and the addition of 0.10 g/kg CYP-Cu in broiler diets is recommended, which suggests that CYP-Cu may be a promising green feed additive in the poultry industry.

## Introduction

*Chinese yam* polysaccharide (CYP) extracted from the *Chinese yam*, a very popular tuber crop for food and medical purposes in China (1), has received attention in recent years, owing to its positive nutritional and medicinal characteristics (2–5). Previous studies have shown that the pharmacological effects of CYP include promoting growth performance, lowering blood glucose and blood lipid, and enhancing anti-oxidation and immunity in animal (6–9). It is known that CYP has good antioxidant activity, and removed hydroxyl radical capacity (4). CYP could enhance the cellular and humoral immune activities in chickens induced by Newcastle disease virus vaccine, and promote proliferation of peripheral blood lymphocytes induced by concanavalin A (9). Another study has shown that mice in a CYP group had heavier body weights than controls, and that CYP could regulate inflammatory responses and oxidative stress in mice (9). Our previous study showed that the supplementation of dietary CYP (0.50 g/kg) promoted thymus index, serum immunoglobulin A (IgA), complement 3 (C3), complement 4 (C4), insulin-like growth factor I (IGF-I), triiodothyronine (T3), thyroxine (T4), insulin (INS), growth hormone (GH), interleukin 2 (IL-2), interleukin 4 (IL-4), and interleukin 6 (IL-6) levels in broilers (10).

An important issue is the application of trace metals in the diets of livestock and poultry. Copper is an essential trace metal in the body, it plays an important role in iron absorption and hemoglobin synthesis (11), and also has strong antimicrobial activity (12). However, inorganic copper is not well absorbed, and can lead to poisoning and environmental pollution, while organic copper is more easily absorbed and used, and reduces environmental pollution and toxic effects (12).

Recent research has shown that the addition of polysaccharides and metal complexes can be effective substances against bacteria (13–15). To date, no published study has evaluated *Chinese yam* polysaccharide copper complex (CYP-Cu) as a dietary additive in animals. In this paper, we prepared CYP-Cu, investigated the effects of its dietary inclusion upon the growth performance, serum immunity levels, and antioxidant capacity in broilers, and discussed the immune function of CYP-Cu in birds, as a strategy to replace antibiotic use and improve growth performance and immunity in poultry.

## Materials and methods

### Preparation of CYP-Cu

The *Chinese yam* polysaccharide for the test was purchased from Shaanxi Hana Biotechnology Co., Ltd (Xi'an city, China), with particle size passing through an 80 mesh sieve (≥95.00%), dry weight loss < 5%, and polysaccharide content ≥30% (total carbohydrate ≥90.40%). The trial copper sulfate was purchased from Tianjin Fenghua Chemical Reagent Factory (analytically pure, Tianjin city, China); molecular formula: CuSO_4_-5H_2_O; molecular weight: 249.68, content: 99.00%. To prepare the CYP-Cu, a water bath was adjusted to 60–70°C; 4 g of CYP and 1 g of sodium citrate were placed into a beaker, and 120 mL of water was added and mixed well; stir until its temperature up to 60–70°C. Sodium hydroxide was added in a dropwise fashion to increase alkalinity; then the saturated copper sulfate solution was added dropwise (to maintain the alkaline environment) until flocculent material appeared; at this point, no more was added. The water bath was stirred for 1 h; the contents were centrifuged while hot, the supernatant was separated and 95% ethanol was added. The solution was left overnight at 4°C, and then centrifuged to produce the precipitate, which is the CYP-Cu. The content of copper in CYP-Cu was determined to be 1591.59 ± 6.07 mg/kg (16).

### Experimental design

In this experiment, 360 1-day-old healthy 817 broilers (39.54 ± 0.51 g, sex balance), a commercial Chinese crossbred broiler produced by crossing a fast-growing broiler cock and layer hen (17), were selected from the same batch at Henan Fengyuan Poultry Co (Xinxiang city, China). The broilers were randomly placed into 4 groups with 3 replicates, with 30 broilers per replicate. The control group was fed only a basal diet (without CYP-Cu); the 3 treated groups were supplemented with 0.02, 0.10, and 0.50 g/kg of CYP-Cu in the basal diet, respectively. The experimental feeding lasted until the chickens were 48 days old, and was divided into two stages, 1–28 and 29–48 days.

### Diets and management

The broilers were fed commercial diets formulated with reference to the NRC (1994) nutritional requirement standards for broilers. The composition and nutritional levels of the basic diet for broilers are shown in Table 1.

**Table 1:** Composition and nutrition level of basic diet.

**Item**	**Content**	
	**1–28 days of age**	**29–48 days of age**
**Ingredient (%)**		
Corn	58.00	63.50
Soybean meal	34.00	29.00
Wheat bran	1.00	
Soybean oil	1.00	2.00
Fish meal	2.00	1.60
Calcium hydrogen phosphate	1.30	1.30
Limestone	1.40	1.30
Table salt	0.30	0.30
Premix^a^	1.00	1.00
Total	100.00	100.00
**Nutrient level**		
Metabolic energy ME (MJ·kg^−1^)^b^	13.23	13.25
Crude protein CP (%)	23.00	20.00
Calcium Ca (%)	1.00	0.90
Total phosphorus TP (%)	0.65	0.6
Available phosphorous AP (%)	0.45	0.35
Lysine met (%)	0.50	0.38
Methionine Lys (%)	1.10	1.00

^a^The premix provided the following per kg of diet: VA 3000 IU, VD_3_ 500 IU, VE 10 IU, VK_3_ 0.5 mg, VB_6_ 3.5 mg, VB_1_ 3.8 mg, D-pantothenic acid 10 mg, folic acid 0.5 mg, biotin 0.15 mg, Fe 80 mg, Cu 8 mg, Zn 75 mg, Mn 60 mg, and Se 0.15 mg.

^b^ME was a calculated value and others were measured values.

All birds were kept under continuous lighting for 24h, and birdhouse temperature was kept at 32°C for the first 3 days, cooled 2–3°C every week, and dropped to 21°C on the 35th until end of the trial. During the experiment, broilers were caged, and had *ad-libitum* access to diet and water; in addition, routine management followed the company's procedures.

### Growth and carcass parameters measured

During the feeding trial period, the amount of intake and leftovers were recorded daily, and body weight (BW), average daily gain (ADG), average daily feed intake (ADFI), and feed conversion ratio (FCR) of broilers were calculated at the end of the trial, respectively. On day 28 and day 48, after collecting blood samples, 6 broilers (3 males and 3 females) in each group were slaughtered, respectively. The data of carcass weight, semi-evisceration weight, evisceration weight, breast muscle, and leg muscle were measured. Then the slaughter percentage (SP), semi-evisceration weight percentage (SEWP), eviscerated carcass weight percentage (EWP), breast muscle percentage (BMP), and leg muscle percentage (LMP) of broilers were determined. The samples of liver tissue were taken from the same position and kept at −80°C until analysis.

### Blood collection

On day 28 and day 48, 6 broilers (3 males and 3 females) were randomly selected from each group for the collection of blood samples. After weighing, the blood was drawn from the wing veins of birds, and put into centrifuge tubes for overnight coagulation at 4°C. Following centrifugation (3,000 g, 10 min, at 4°C), the serum was collected into sterile tubes and kept at −80°C until analysis.

### Serum biochemical, immune, and antioxidative parameters measured

Serum biochemistry parameters, such as serum albumin (ALB), low density lipoprotein (LDL), and high density lipoprotein (HDL), were obtained by using a 7,180 automatic analyzer (Hitachi High-Technologies Co., Ltd., Tokyo, Japan). The assays of serum biochemical, immune, and antioxidative profiles included insulin-like growth factor I (IGF-I), triiodothyronine (T3), thyroxine (T4), growth hormone (GH), insulin (INS), immunoglobulin M (IgM), immunoglobulin G (IgG), immunoglobulin A (IgA), interleukin 2 (IL-2), interleukin 4 (IL-4), interleukin 6 (IL-6), complement 3 (C3), complement 4 (C4), total superoxide dismutase (T-SOD), total antioxidative capacity (T-AOC), malondialdehyde (MDA), glutathione peroxidase (GSH-Px), and glutathione s-transferase (GSH-ST); concentrations were analyzed by ELISA according to the manufacturer's instruction (Nanjing Jiancheng Bioengineering Institute, Nanjing city, China).

### Assay of hepatic antioxidative gene expressions

Total RNAs were isolated from hepatic tissues using Trizol reagent (Invitrogen, Thermo Fisher Scientific, USA) following the manufacturer's instructions. The quality and quantity of RNA was detected with a NanoPhotometer^®^ spectrophotometer (Implen, Westlake Village, CA, USA), and RNA integrity wasdetected with 1.0% agarose gelelectrophoresis. Total RNAs were transcribed into cDNA using M-MLV reverse transcriptase (Promega, Madison, WI, USA). The genes of Nuclear factor E2-related factor 2 (*Nrf*_2_), Superoxide dismutase 1 (*SOD*_1_), Superoxide dismutase 2 (*SOD*_2_), and Catalase (*CAT*) were selected for the detection of hepatic antioxidative gene expression profiles. The primer sequences for quantitative real-time PCR (qRT-PCR) assay are shown in Table 2. The qRT-PCR was carried out in a QuantStudio 6 Flex Real-Time PCR System (ABI, Carlsbad, CA, USA) with the miScript SYBR Green PCR kit (Qiagen, GmbH, Hilden, Germany) following the manufacturer's instructions. In the present study, β-actin as a housekeeping gene, the relative expression of the target genes was calculated by 2^−ΔΔCt^ method.

**Table 2:** Primer design for hepatic antioxidant gene assay by qRT-PCR.

**Gene**	**Primer sequence (5^′^-3^′^)**	**Product length (bp)**
*β-actin*	F:5**′**-CATTGAACACGGTATTGTCACCAACTG-3**′**	270
	R:5**′**-GTAACACCATCACCAGAGTCCATCAC-3**′**	
*Nrf_2_*	F:5**′**-AACACACCAAAGAAAGACCCTCCTG-3**′**	207
	R:5**′**-TTCACTGAACTGCTCCTTCGACATC-3**′**	
*SOD_1_*	F:5**′**-GGTCATCCACTTCCAGCAGCAG-3**′**	377
	R:5**′**-AACGAGGTCCAGCATTTCCAGTTAG-3**′**	
*SOD_2_*	F:5**′**-TTCCTGACCTGCCCTACGACTATG-3**′**	357
	R:5**′**-AGCCTGATCCTTGAACACCAACTG-3**′**	
*CAT*	F:5**′**-CTCTCAGAAGCCAGATGCCTTGAC-3**′**	293
	R:5**′**-CAGCAACAGTGGAGAACCGTATAGC-3**′**	

Nrf_2_, nuclear factor E2-related factor 2; SOD_1_, superoxide dismutase 1; SOD_2_, superoxide dismutase 2; CAT, catalase.

### Statistical analysis

All data obtained from this experiment were analyzed using One-Way ANOVA in SPSS 26.0 for Windows (IBM Corp., Chicago, IL, USA), and presented as mean ± standard error of the means (SEM), and significant differences among all groups were determined at *P* < 0.05 by Duncan's multiple range tests.

## Results

### Growth performances

The effect of dietary CYP-Cu on growth performances evaluated in this study is shown in Table 3. The BW of broilers on day 28 and day 48 was affected by the addition of CYP-Cu, the BW of the 0.10 and 0.50 g/kg CYP-Cu group was significantly higher than that of the control group on day 28 (*P* < 0.05), while the BW of the 0.10 g/kg group was significantly higher than that of control group on day 48 (*P* < 0.05). Compared with the control, the ADG of the treatments increased with the addition of CYP-Cu, and the 0.10 g/kg group had the highest ADG in all trial periods (*P* < 0.05). However, no difference of ADFI between the treatments and the control were observed in all trial periods (*P* > 0.05). By contrast, the FCR of the treatments in 29–48 days periods was significantly lower than that of control group (*P* < 0.05), while no difference of FCR between the treatments and the control were observed in 1–28 and 1–48 days periods (*P* > 0.05). However, the FCR of the treatments had a downward trend with the addition of CYP-Cu, and the 0.10 g/kg group had the lowest FCR in all trial periods.

**Table 3:** Effect of dietary CYP-Cu on the growth performance in broilers.

**CYP-Cu level**							
**Item**	**Age**	**0 g/kg**	**0.02 g/kg**	**0.10 g/kg**	**0.50 g/kg**	**SEM**	***P*** **-value**
BW (g)	1 day	39.71	39.86	39.67	39.70	0.437	0.972
	28 days	618.11^b^	630.10^ab^	638.63^a^	640.49^a^	5.702	0.016
	48 days	1,560.53^c^	1,623.61^b^	1,682.35^a^	1,658.08^ab^	22.067	0.003
ADFI (g/days)	1–28 days	31.93	31.34	31.25	31.59	0.528	0.597
	29–48 days	98.16	97.48	99.31	98.56	1.831	0.791
	1–48 days	60.41	61.60	62.87	62.81	1.413	0.321
ADG (g/days)	1–28 days	20.66^b^	21.08^ab^	21.46^a^	21.39^a^	0.207	0.017
	29–48 days	47.12^b^	49.68^a^	52.09^a^	50.97^a^	1.064	0.008
	1–48 days	31.68^c^	33.00^b^	34.22^a^	33.72^ab^	0.460	0.003
FCR	1–28 days	1.54	1.48	1.45	1.47	0.035	0.138
	29–48 days	2.18^a^	1.96^b^	1.91^b^	1.93^b^	0.030	0.002
	1–48 days	1.91	1.87	1.84	1.86	0.027	0.165

CYP-Cu, *Chinese yam* polysaccharide copper complex; SEM, standard error of the means (n = 6); BW, body weight; ADFI, average daily feed intake; ADG, average daily gain; FCR, feed conversion ratio.

In the same line, values with different lowercase superscripts indicate significant differences (*P* < 0.05), and values with the same lowercase superscripts indicate no significant differences (*P* > 0.05).

### Carcass performances

The supplementation of dietary CYP-Cu had a positive effect on the carcass performances of broilers on day 48 (Table 4). Compared to that of the control, SP, SEWP, EWP, BMP, and LMP of 48-day broilers showed a gradual increase with the addition of CYP-Cu. Among them, the carcass performances of the 0.10 and 0.50 g/kg CYP-Cu group showed a significantly greater increase than those of the control in 48-day broilers (*P* < 0.05).

**Table 4:** Effect of dietary CYP-Cu on the carcass performance in broilers on day 48.

**CYP-Cu level**							
**Item**	**Age**	**0 g/kg**	**0.02 g/kg**	**0.10 g/kg**	**0.50 g/kg**	**SEM**	***P*** **-value**
SP (%)	48 days	88.21^b^	89.96^a^	90.75^a^	90.03^a^	0.560	0.011
SEWP (%)	48 days	80.56^b^	81.84^a^	82.36^a^	81.79^a^	0.389	0.010
EWP (%)	48 days	67.70^b^	68.14^ab^	69.10^a^	68.51^a^	0.374	0.030
BMP (%)	48 days	17.78^b^	18.28^ab^	18.69^a^	18.37^a^	0.307	0.022
LMP (%)	48 days	20.75^c^	21.38^b^	22.22^a^	21.63^b^	0.240	0.002

CYP-Cu, *Chinese yam* polysaccharide copper complex; SEM, standard error of the means (n = 6); SP, slaughter percentage; SEWP, semi-evisceration weight percentage; EWP, eviscerated carcass weight percentage; BMP, breast muscle percentage; LMP, leg muscle percentage.

In the same line, values with different lowercase superscripts indicate significant differences (*P* < 0.05), and values with the same lowercase superscripts indicate no significant differences (*P* > 0.05).

### Serum growth factors and biochemical parameters

As presented in Table 5, the concentrations of serum growth factors, IGF-I, T3, T4, GH, and INS, in the 3 treated groups expressed a higher tendency than those in the control group of broilers on day 28 and day 48. The results of the 0.10 g/kg CYP-Cu group were the most significant (*P* < 0.05), with the exception of serum GH concentration in the 0.50 g/kg group, which had the highest level on day 28 (*P* < 0.05).

**Table 5:** Effects of dietary CYP-Cu on serum growth factors and biochemical parameters in broilers.

**CYP-Cu level**							
**Item**	**Age**	**0 g/kg**	**0.02 g/kg**	**0.10 g/kg**	**0.50 g/kg**	**SEM**	***P*** **-value**
ALB (g/L)	28 days	22.05^b^	24.51^a^	25.35^a^	24.96^a^	0.467	0.002
	48 days	20.83^c^	23.19^ab^	23.60^a^	22.57^b^	0.356	0.001
LDL (μmol/L)	28 days	378.65	375.89	353.56	369.70	3.730	0.312
	48 days	401.15	401.74	392.64	388.22	6.873	0.216
HDL (μmol/L)	28 days	131.83^d^	140.31^c^	145.87^b^	151.71^a^	1.641	0.004
	48 days	129.05^b^	147.04^a^	147.50^a^	145.99^a^	3.023	0.001
IGF-1 (μg/L)	28 days	36.17^c^	39.30^b^	42.61^a^	37.01^c^	0.633	0.001
	48 days	34.80^b^	39.29^a^	40.20^a^	37.82^a^	1.254	0.012
T3 (pmol/L)	28 days	288.39^d^	307.14^c^	363.10^a^	340.15^b^	4.215	0.007
	48 days	297.92^c^	316.37^b^	346.50^a^	307.69^bc^	4.532	0.009
T4 (pmol/L)	28 days	928.44^c^	950.19^bc^	1,116.95^a^	990.52^b^	19.075	0.004
	48 days	1,025.39^c^	1,041.74^c^	1,181.25^a^	1,093.74^b^	13.518	0.015
GH (μg/L)	28 aysd	18.24^d^	20.29^c^	20.94^b^	21.69^a^	0.232	0.002
	48 days	20.43^b^	24.49^a^	25.45^a^	24.84^a^	0.550	0.003
INS (μg/L)	28 days	16.35^c^	17.50^b^	20.42^a^	18.14^b^	0.375	0.003
	48 days	16.78^c^	19.53^a^	19.85^a^	18.90^b^	0.268	0.005

CYP-Cu, *Chinese yam* polysaccharide copper complex; SEM, standard error of the means (n = 6); ALB, serum albumin; LDL, low density lipoprotein; HDL, high density lipoprotein; IGF-I, insulin-like growth factor I; T3, triiodothyronine; T4, thyroxine; GH, growth hormone; INS, insulin.

In the same line, values with different lowercase superscripts indicate significant differences (*P* < 0.05), and values with the same lowercase superscripts indicate no significant differences (*P* > 0.05).

According to Table 5, the densities of ALB and HDL in the 3 treated groups were significantly higher than those in the control group on day 28 and day 48 (*P* < 0.05). ALB levels in the 0.10 g/kg CYP-Cu group were highest in broilers on day 28 and day 48 (*P* < 0.05), while HDL level was highest in the 0.50 g/kg CYP-Cu group on day 28 and in the 0.10 g/kg CYP-Cu group on day 48 (*P* < 0.05). Interestingly, the densities of LDL in the 3 treated groups had a decreasing trend with increasing dietary CYP-Cu supplementation, but there were no significant differences in LDL levels between the treated and the control groups (*P* > 0.05).

### Serum immune parameters

The serum immune parameters in broilers on day 28 and day 48 are shown in Table 6. Compared with the control group, the serum levels of IgM, IgA, IgG, IL-2, IL-4, IL-6, C3, and C4 in the 3 treated groups were significantly higher on day 28 (*P* < 0.05). The dietary supplementations of 0.10 and 0.50 g/kg CYP-Cu were more effective. On day 48, the serum concentrations of IgM, IgA, IgG, IL-2, IL-6, C3, and C4 in the 3 treated groups were significantly higher than those in the control group (*P* < 0.05), meanwhile, the serum levels of IL-2 in the 0.02 and 0.10 g/kg CYP-Cu groups were significantly higher than those in the control group (*P* < 0.05). The additions of 0.10 g/kg CYP-Cu in the diet of broilers on day 48 seemed to have a greater effect on serum immune parameters.

**Table 6:** Effects of dietary CYP-Cu on serum immune parameters in broilers.

**CYP-Cu level**							
**Item**	**Age**	**0 g/kg**	**0.02 g/kg**	**0.10 g/kg**	**0.50 g/kg**	**SEM**	***P*** **-value**
IgM (ng/mL)	28 days	5,866.38^c^	6,255.93^b^	6,607.24^a^	6,363.59^b^	90.639	0.034
	48 days	5,130.01^d^	5,774.12^c^	6,312.90^a^	5,954.52^b^	77.100	0.020
IgA (ng/mL)	28 days	8,588.37^b^	9,779.10^a^	9,886.84^a^	9,783.41^a^	105.803	0.005
	48 days	8,158.15^c^	8,533.61^b^	9,284.54^a^	8,617.64^b^	155.892	0.001
IgG (μg/mL)	28 days	85.98^c^	92.05^b^	93.20^b^	98.64^a^	1.380	0.001
	48 days	79.05^d^	84.50^c^	88.99^b^	92.75^a^	1.490	0.003
IL-2 (ng/L)	28 days	143.34^b^	167.42^a^	168.06^a^	170.12^a^	2.831	0.013
	48 days	179.47^d^	224.00^b^	235.09^a^	211.96^c^	3.287	0.000
IL-4 (ng/L)	28 days	168.19^b^	213.92^a^	214.88^a^	213.27^a^	2.406	0.002
	48 days	169.74^c^	180.27^ab^	182.20^a^	172.06^bc^	3.827	0.029
IL-6 (ng/L)	28 days	40.78^c^	52.55^b^	52.99^b^	56.06^a^	0.884	0.011
	48 days	42.65^c^	52.71^b^	55.24^a^	51.81^b^	0.895	0.035
C3 (μg/mL)	28 days	713.88^b^	802.75^a^	824.36^a^	814.49^a^	14.527	0.001
	48 days	725.93^c^	824.37^b^	859.78^a^	848.45^ab^	14.146	0.006
C4 (μg/mL)	28 days	481.07^c^	502.76^b^	560.58^a^	567.36^a^	8.599	0.007
	48 days	457.13^c^	489.59^b^	570.52^a^	511.79^b^	10.197	0.001

CYP-Cu, *Chinese yam* polysaccharide copper complex; SEM, standard error of the means (n = 6); IgM, immunoglobulin M; IgA, immunoglobulin A; IgG, immunoglobulin G; IL-2, interleukin 2; IL-4, interleukin 4; IL-6, interleukin 6; C3, complement 3; C4, complement 4.

In the same line, values with different lowercase superscripts indicate significant differences (*P* < 0.05), and values with the same lowercase superscripts indicate no significant differences (*P* > 0.05).

### Serum antioxidative parameters

According to Table 7, the dietary supplementation of CYP-Cu played a positive role in serum antioxidative parameters in broilers on day 28 and day 48. Compared with that of the control, the serum concentrations of T-SOD, T-AOC, GSH-ST, and GSH-Px in the treated groups were higher on day 28 and day 48. Therein, the results of the 0.10 and 0.50 g/kg CYP-Cu groups were the most significant on day 28 (*P* < 0.05), while the results of the 0.50 g/kg CYP-Cu group seemed to be more effective on day 48 (*P* < 0.05). Interestingly, the serum concentration of MDA in the treated groups was downregulated in contrast to the control on day 28 and day 48. The serum concentration of MDA of the 0.10 and 0.50 g/kg CYP-Cu groups showed a greater decline (*P* < 0.05). These results indicated that the supplementation of CYP-Cu in the diet of broilers could raise oxidation resistance.

**Table 7:** Effects of dietary CYP-Cu on serum antioxidative parameters in broilers.

**CYP-Cu level**							
**Item**	**Age**	**0 g/kg**	**0.02 g/kg**	**0.10 g/kg**	**0.50 g/kg**	**SEM**	***P*** **-value**
T-SOD (pg/mL)	28 days	41.15^c^	46.18^b^	49.94^a^	47.02^b^	0.830	0.006
	48 days	44.07^d^	49.08^b^	50.52^a^	47.60^c^	0.609	0.025
T-AOC (U/mL)	28 days	5.26^c^	5.74^b^	5.88^b^	6.24^a^	0.107	0.012
	48 days	5.20^b^	5.93^a^	5.94^a^	6.19^a^	0.123	0.014
MDA (nmol/L)	28 days	15.24^a^	15.01^a^	13.21^b^	14.53^a^	0.359	0.002
	48 days	15.88^a^	15.28^b^	14.70^c^	14.98^bc^	0.218	0.004
GSH-Px (pmol/mL)	28 days	12.72^c^	14.13^b^	14.66^a^	14.73^a^	0.214	0.001
	48 days	15.22^b^	17.56^a^	17.67^a^	17.99^a^	0.211	0.001
GSH-ST (ng/L)	28 days	462.53^c^	466.12^c^	497.23^b^	520.47^a^	7.321	0.001
	48 days	471.30^b^	476.49^b^	481.52^b^	510.79^a^	8.704	0.008

CYP-Cu, *Chinese yam* polysaccharide copper complex; SEM, standard error of the means (n = 6); T-SOD, total superoxide dismutase; T-AOC, total antioxidative capacity; MDA, malondialdehyde; g GSH-Px, lutathione peroxidase; GSH-ST, glutathione s-transferase.

In the same line, values with different lowercase superscripts indicate significant differences (*P* < 0.05), and values with the same lowercase superscripts indicate no significant differences (*P* > 0.05).

### Hepatic antioxidative gene expression profiles

**Figure 1:**
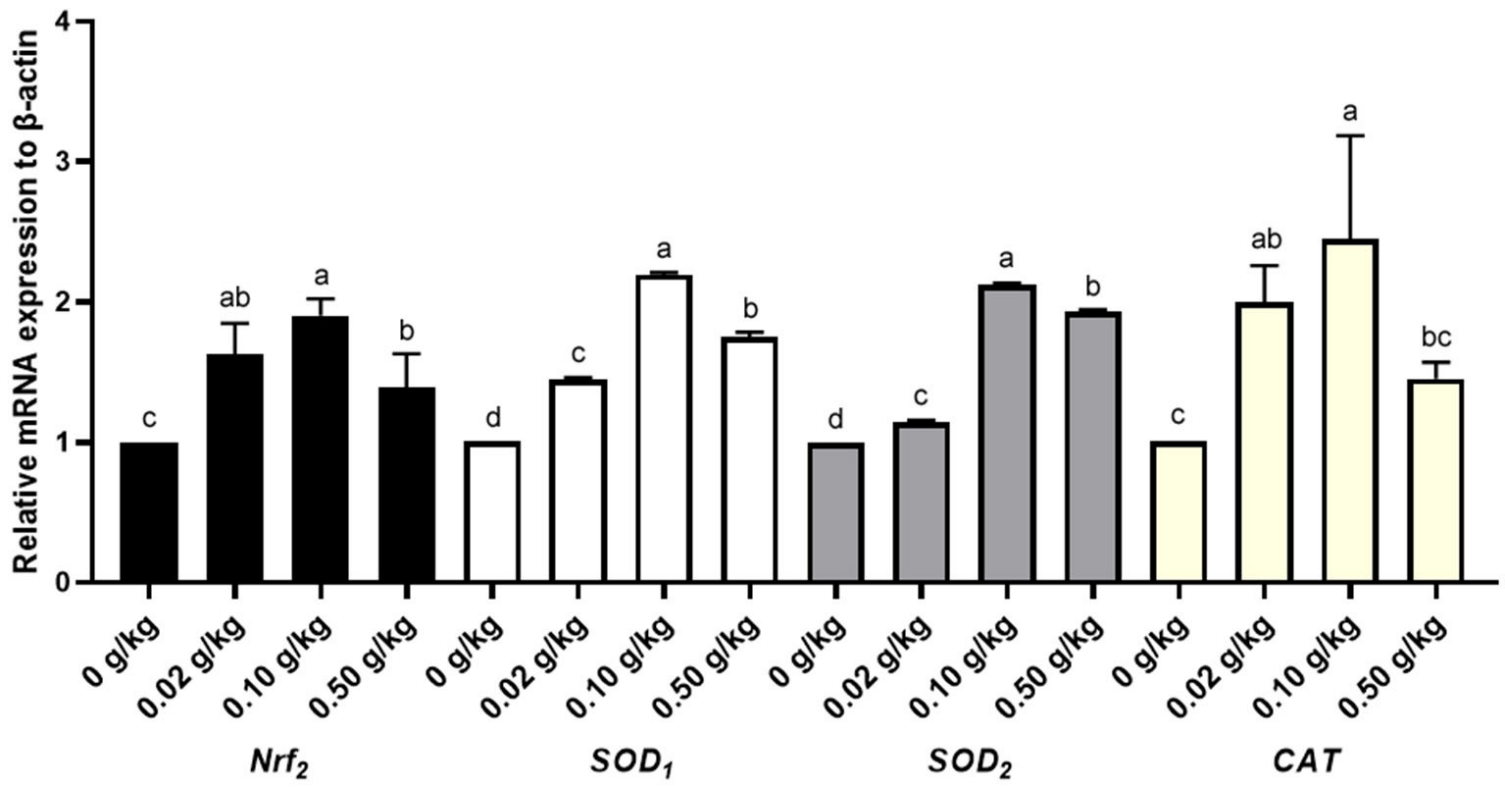
Hepatic antioxidative gene expression profiles in 48-day-old broilers. *Nrf*_2_, nuclear factor E2-related factor 2; *SOD*_1_, superoxide dismutase 1; *SOD*_2_, superoxide dismutase 2; *CAT*, catalase. In the figure, antioxidative gene expression profiles of *Nrf*_2_, *SOD*_1_, *SOD*_2_, and *CAT*, in the livers of 48-day-old broilers were detected by qRT-PCR. The values with different lowercase superscripts indicate significant differences (*P* < 0.05), while values with the same lowercase superscripts indicate no significant differences (*P* > 0.05).

In this experiment, the antioxidant genes, *CAT, Nrf*_2_, *SOD*_1_, *SOD*_2_, and mRNA expression in the livers of broilers on day 48 were investigated (Figure 1, Supplementary Table S1). According to Figure 1, the antioxidative gene mRNA expression of *Nrf*_2_, *SOD*_1_, *SOD*_2_ and *CAT* were significantly upregulated in the liver in the treated groups compared to the control group (*P* < 0.05), while among all the treated groups, the antioxidative genes had the highest mRNA expression level in the 0.10 g/kg group. The results show that the dietary supplementation of 0.10 g/kg CYP-Cu could improve mRNA expression of the antioxidant genes *CAT, Nrf*_2_, *SOD*_1_, and *SOD*_2_ in the livers of broilers on day 48, and enhance their antioxidant capacity.

## Discussion

In recent years, increasing numbers of studies have shown that polysaccharide metal complexes display diverse biological activities, such as improving growth, antioxidation, immune regulation, and antibacterial activity (14, 18–21). The objective of this paper was to determine the effect of supplementation of CYP-Cu in broiler diets on growth performance, immune function, and antioxidant capacity. The treated results showed that such supplementation improved the growth and carcass performance of broiler chickens, and a dietary supplementation of 0.10 g/kg CYP-Cu was the optimal level. Cui et al. (22) reported that the supplementation of *Enteromorpha prolifera* polysaccharide-iron (III) complex (2 mg Fe/kg body weight) by intragastric administration has a considerable effect on the growth performance and blood indexes of rats with iron deficiency anemia, indicating that *Enteromorpha prolifera* polysaccharide-iron (III) complex could be used as a new iron fortifier to replace traditional iron supplements for gastrointestinal irritation and poor absorption. Another study reported the effects of pectin oligosaccharide and zinc chelate (80 mg/kg) on growth performance, antioxidant ability, and gut function in broilers, and found that dietary supplementation with pectin oligosaccharide and zinc chelate was more effective than feeding pectin oligosaccharide and zinc sulfate alone (23). Gao et al. (20) showed that soybean polysaccharide iron complex (5.00–20.00 mg/mL) had different impacts on foodborne bacteria, promoting the growth of the beneficial bacteria, *Bacillus licheniformis*, and inhibiting the proliferation of the pathogenic bacteria, *Staphylococcus aureus*, thus improving the body's growth and immunity. These studies indicate that, compared with polysaccharide and metal alone, polysaccharide and metal complexes improve animal growth and immunity because of the synergistic interaction between polysaccharides and metals, and the enhancement of intestinal health and nutrient absorption by promoting the proliferation of beneficial bacteria to the competitive exclusion of pathogenic bacteria. Consistent with previous studies, our results showed that CYP-Cu had a positive effect on growth performance of broilers, implying a good synergy.

In the present study, the addition of CYP-Cu in diet can enhance the immunity of broilers by improving the concentrations of IgM, IgA, IgG, IL-2, IL-4, IL-6, C3, and C4 in serum at 28 days and in serum IgM, IgA, IgG, IL-2, IL-6, C3, and C4 in group 48 days, and we recommend the supplementation of 0.10 g/kg CYP-Cu in broiler diets. However, little is known about the underlying principle by which dietary CYP-Cu improves the immune performance of broilers. Gao et al. (19) demonstrated that Ulva polysaccharide iron complex (200 and 400 mg/mL) could promote the proliferation of lymphocytes, improve the activities of murine macrophages, and restore serum levels of IFN-γ, and IL-10 in immune-deficient mice to normal; furthermore, the Ulva polysaccharide iron complex exhibits excellent hematopoietic capacity. Dey et al. (24) found that chitosan conjugated green copper oxide nanoparticles (50 μg/mL) inhibited the proliferation of breast cancer and cervical cancer cells *in vivo* in a Balb/C mouse model, and increased pro-inflammatory cytokines and CD^4+^ populations; the author showed that the potential mechanism of chitosan conjugated green copper oxide nanoparticles is not only through inducing cellular immunity by activating immune cells, but they also lead to a humoral immune response through an IgG reaction. Another study reports that copper-loaded chitosan nanoparticles (100 mg/kg) in broiler diets improves the growth of poultry, and raises the serum levels of IgA, IgM, C3, and C4, suggesting that it can be used as a substitute for antibiotics (25). Although previous studies have exhibited the immune activities of polysaccharide metal complexes in animals, further research is needed to explain the underlying mechanisms by which dietary CYP-Cu improves immune performance in animals.

Free radicals, *in vivo*, are products that are inevitably generated through metabolism in a broad range of biochemical reactions; when accumulation is excessive they can lead to oxidative stress and subsequent tissue damage (26–28). Antioxidants are the substances that protect organisms against damage caused by oxidation, and they are classified into two groups, exogenous and endogenous antioxidants, according to origin. To date, increasing numbers of studies indicate that natural botanical polysaccharides are potential antioxidants (29, 30). In this paper, an evaluation about the effects of the supplementation of dietary CYP-Cu on serum antioxidative parameters of broilers demonstrated that dietary CYP-Cu significantly increased the serum concentrations of T-SOD, T-AOC, GSH-ST, GSH-Px, and decreased the serum concentrations of MDA of broilers (0.10 g/kg CYP-Cu group), which revealed that CYP-Cu had strong antioxidant activity. A previous study shown that lotus root polysaccharide iron complex could significantly increase the antioxidant capacity of mice by increasing the serum levels of CAT, SOD, and GSH-Px (31), and showed a trend of first increasing from 2 to 8 mg/mL and then decreasing. Similarly, Dong et al. (32) found that Flammulina velutipes polysacchrides and polysacchride-iron (III) complex inhibited the MDA production of health mice liver and improve its antioxidant capacity. Li et al. (33) found that novel biochanin a-chromium (III) complex enhanced the oxidative capacity of the *db*/*db* mice and improved the oxidative stress injury caused by hyperglycemia through decreasing the content of MDA, and increasing the content of CAT, SOD, and GSH-Px in liver of mice. Our study also demonstrated that dietary CYP-Cu could improve the antioxidant capacity of broilers.

On the other hand, the current study showed that the supplementation of dietary CYP-Cu in broilers had the ability to upregulate the mRNA expression of hepatic antioxidant genes, *CAT, Nrf*_2_, *SOD*_1_, and *SOD*_2_, and the dietary supplementation of 0.10 g/kg CYP-Cu in broilers was more effective in promoting antioxidant gene expressions. Gene expressions of antioxidant enzymes such as SOD, glutathione peroxidase, and heme oxygenase-1 are the key mechanism by which the body responds to various reactive oxygen species during inflammation, trauma, or other stressful conditions (34). SODs are a family of metalloproteins that catalyze the change of radical superoxide to oxygen and hydrogen peroxide, and protect organisms against oxidative stress. SOD_1_ and SOD_2_, such as Cu–Zn SOD and Mn SOD, are located in the cytoplasm and mitochondria (35, 36). Chinese wolfberry and Astragalus extract (1%) notably improved the activities of SOD_1_, T-AOC, and CAT, and decreased MDA in Tibetan pig livers, and promoted the expression levels of hepatic antioxidant genes (e.g., *CAT* and *SOD*_1_); this indicates that the extracts mechanism of regulating antioxidation is the signaling pathway of peroxisome antioxidant-oxidant stress in Tibetan pig livers (37). Another study investigated the effect of a plateau condition on oxidation in Tibetan pigs and Duroc × Landrace × Yorkshire (DLY) pigs, and found that Tibetan pigs exhibited higher SOD, GSH-Px, and T-AOC levels, but lower MDA levels in the liver and heart. Furthermore, the mRNA levels of *SOD, GSH-Px*, and *Nrf*_2_ in the liver and heart of Tibetan pigs were higher than those in DLY pigs, and the authors suggest that Tibetan pigs held a stronger antioxidant activity through the AMPK/p38 MAPK/Nrf_2_-ARE signaling pathways (38). Wang and Li (18) found that the Zinc-HSP (*Hohenbuehelia serotina* polysaccharides) complex (20 mg/mL) not only had superoxide anion radical scavenging ability, but also did not affect the biological activity of *Hohenbuehelia serotina* polysaccharides when combined with zinc. The dietary supplementation of *Enteromorpha prolifera* polysaccharide-zinc (EP-Zn) complex (400 mg EP-Zn/kg) in chickens upregulated the mRNA expression levels of the antioxidant genes *Nrf*_2_, *CAT*_1_, and *SOD*_1_ in breast muscle and lipid metabolism genes *ACC, FADS*_1_, *PPAR-*α, and *CPT*_1_ in liver, thereby improving the growth performance and meat quality of chickens (39). In this paper, dietary supplementation of CYP-Cu has been shown to have effective free radical scavenging and antioxidant activities in broilers, which indicates that CYP-Cu has great potential for the application in poultry.

## Conclusion

The current results showed that dietary CYP-Cu improved growth performance, serum immune function, and antioxidant capacity in broilers, and upregulated the hepatic antioxidant gene *CAT, Nrf*_2_, *SOD*_1_, and *SOD*_2_ mRNA expression of broilers, and the supplementation of 0.10 g/kg CYP-Cu in broiler diet is recommended.

## Data availability statement

The original contributions presented in the study are included in the article/Supplementary material, further inquiries can be directed to the corresponding author.

## Ethics statement

The animal study was reviewed and approved by the Animal Protection and Utilization Committee of Henan Institute of Science and Technology (No. 2021HIST018, Xinxiang, P. R. China).

## Author contributions

JZ: design, investigation, writing—original manuscript, and editing. YJ, MC, JD, and YC: carried out the experiments and analyzed the data. MS: conceptualization, methodology, and supervision. ZM: project administration and funding acquisition. All authors contributed to the article and approved the submitted version.
