# Supplementary material for: Effects of dietary Chinese yam polysaccharide copper complex on growth performance, immunity, and antioxidant capacity of broilers
Source: Front Vet Sci. 2023 Feb 16;10:1123002. doi: 10.3389/fvets.2023.1123002 (PMC9978188; doi:10.3389/fvets.2023.1123002)
Supplement: Supplementary file 1 [file Table_1.docx]

**Table S1.** *CAT*, *Nrf_2_*, *SOD_1_*, *SOD_2_* mRNA expression profiles in liver of 48-day-old broilers

| CYP-Cu level (g/kg)^1^ | | | | | | | |
| --- | --- | --- | --- | --- | --- | --- | --- |
| Item | Age | Control | CYP-Cu I | CYP-Cu II | CYP-Cu III | SEM | *P*-value |
| *CAT* | 48d | 0.46^b^ | 0.92^ab^ | 1.13^a^ | 0.67^ab^ | 0.153 | 0.011 |
| *SOD_1_* | 48d | 0.73^c^ | 1.06^b^ | 1.60^a^ | 1.27^b^ | 0.024 | 0.000 |
| *SOD_2_* | 48d | 0.43^c^ | 0.49^c^ | 0.91^a^ | 0.83^b^ | 0.007 | 0.000 |
| *Nrf_2_* | 48d | 0.72^c^ | 1.17^ab^ | 1.37^a^ | 1.00^bc^ | 0.108 | 0.002 |

In the same line, values with different lowercase superscripts indicate significant differences (P < 0.05), and values with the same lowercase superscripts indicate no significant differences (P > 0.05). ^1^Control, CYP-Cu I, CYP-Cu II, and CYP-Cu III represented the data of broilers fed with 0 g/kg, 0.02 g/kg, 0.10 g/kg, and 0.50 g/kg in the diet, respectively.
